# Precision Genomics: A Reality Having Universal Impact in a New Era of Psychiatry – Lessons Learned, Past and Present

**DOI:** 10.17756/jap.2025-050

**Published:** 2025-08-29

**Authors:** Kenneth Blum, Alireza Sharafshah, Jag Khalsa, Kai Uwe-Lewandrowski, Kavya Mohankumar, Panayotis K. Thanos, Albert Pinhasov, David Baron, Catherine A. Dennen, Joseph J. Morgan, Marco Lindenau, Igor Elman, Eliot L. Gardner, Mark S. Gold, Edward J. Modestino, Fuehrlein Brian, Paul R. Carney, Rene Cortese, Abdalla Bowirrat, Margaret A. Madigan, Keerthy Sunder, Morgan P. Lorio, Foojan Zeine, Nicole Jafari, Milan T. Makale, Debasis Bagchi, Mauro Ceccanti, Rossano K.A. Fiorelli, Sérgio Luís Schimidt, Daniel Sipple, Alexander P.L. Lewandrowski, Gianni Matare, Shaurya Mahajan, Yatharth Mahajan, Colin Hanna, Daniel Gastelu, Anand Swaroop, Chynna Fliegelman, Rajendra D. Badgaiyan

**Affiliations:** 1 Department of Molecular Biology, Adelson School of Medicine, Ariel University, Ariel, Israel; 2 Division of Addiction Research & Education, Center for Sports, Exercise, and Mental Health, Western University of Health Sciences, Pomona, USA; 3 Division of Clinical Neurology, The Blum Institute of Neurogenetics & Behavior, Austin, USA; 4 Cellular and Molecular Research Center, School of Medicine, Guilan University of Medical Sciences, Rasht, Iran; 5 Department of Medicine, School of Medicine, University of Maryland, Baltimore, USA; 6 Division of Personalized Pain Therapy Research & Education, Center for Advanced Spine Care of Southern Arizona, Tucson, USA; 7 Department of Orthopaedics, Sanitas University Foundation Bogotá, Colombia; 8 Department of Orthopaedics, Federal University of the State of Rio de Janeiro, Brazil; 9 Department of Surgery, Arizona University School of Medicine, Tucson, USA; 10 Department of Pharmacology and Toxicology, Jacobs School of Medicine and Biosciences, State University of New York at Buffalo, Buffalo, USA; 11 Department of Psychiatry, Stanford University School of Medicine, Palo Alto, USA; 12 Department of Family Medicine, Jefferson Health Northeast, Philadelphia, USA; 13 Substance Use Disorders Institute University of Sciences, Philadelphia, USA; 14 Department of Psychiatry and Cambridge Health Alliance, Harvard Medical School, Cambridge, USA; 15 Department of Psychiatry and Behavioural Sciences, Johns Hopkins University School of Medicine, Baltimore, USA; 16 Department of Psychiatry, Washington University School of Medicine, USA; 17 Department of Psychology, Curry College, Milton, USA; 18 Department of Psychiatry, Yale University, New Haven, USA; 19 Division Pediatric Neurology, School of Medicine, University of Missouri, Columbia, USA; 20 Department of Child Health, Child Health Research Institute, School of Medicine, University of Missouri, Columbia, USA; 21 Department of Medicine, Riverside School of Medicine, University of California, Riverside, USA; 22 Karma Doctors & Karma TMS and Suder Foundation, Palm Springs, USA; 23 Orlando College of Osteopathic Medicine (OCOM), Orlando, USA; 24 Awareness Integration Institute, San Clemente, USA; 25 Department of Health & Sciences, California State University Long Beach, Long Beach, USA; 26 Division of Personalized Medicine, Global Growth Institute, Inc., San Clemente, USA; 27 Department of Applied Clinical Psychology, The Chicago School of Professional Psychology, USA; 28 Department of Radiation Oncology, University of California San Diego, La Jolla, USA; 29 Department of Pharmaceutical Sciences, Texas Southern University College of Pharmacy, Houston, USA; 30 Alcohol Addiction Program, Latium Region Referral Center, Sapienza University of Rome, Roma, Italy; 31 Department of General and Specialized Surgery, Gaffrée e Guinle Universitary Hospital (EBSERH), Federal University of the State of Rio de Janeiro (UNIRIO), Rio de Janeiro, Brazil; 32 Graduate Program in Neurology, Federal University of the State of Rio de Janeiro, Rio de Janeiro, Brazil; 33 M Health Fairview University of Minnesota Medical Center, Minneapolis, USA; 34 Dornsife College of Letters, Arts and Sciences, University of Southern California, Los Angeles, USA; 35 Department of Psychology, University South Dakota, Vermillion, USA; 36 Cepham Inc., Summerset, New Jersey, USA; 37 Department of Psychology, St. John’s University, Queens, USA; 38 Department of Psychiatry, Texas Tech University Health Sciences, Lubbock, USA; 39 Department of Psychiatry, Mt. Sinai University, School of Medicine, New York, USA

**Keywords:** Genomics, Psychiatry, Disorder, Syndrome

## Abstract

Addiction neuroscience explores the complex interplay between genetic, neurobiological, environmental, and socio-spiritual factors underlying substance and behavioral addictions. Over the past three decades, research in this domain has identified critical molecular and epigenetic mechanisms—particularly those affecting dopaminergic signaling and reward pathways—that contribute to both vulnerability and resilience to addictive behaviors. Central to this understanding is the concept of reward deficiency syndrome (RDS), first introduced by Kenneth Blum, which posits that hypodopaminergic functioning predisposes individuals to seek maladaptive rewards. Advances in neurogenetics, including the identification of key polymorphisms such as the DRD2 A1 allele, have paved the way for precision tools like the genetic addiction risk severity (GARS^®^) test. This test, alongside pro-dopaminergic nutraceutical interventions like KB220, demonstrates the potential for early detection and individualized treatment of “pre-addiction” risk states. Despite ongoing reliance on opioids for opioid use disorder (OUD), emerging paradigms advocate for dopamine homeostasis through non-addictive, integrative approaches. Furthermore, the integration of whole genome sequencing data can be used for Genome-Wide Association Studies (GWAS), multi-omics, and machine learning into clinical practice holds promise for advancing personalized medicine in addiction treatment. As the field progresses, addressing health equity and improving genomic representation across populations remain critical goals. This evolving framework underscores the importance of leveraging genomic insights to prevent, predict, and personalize interventions for addiction and mental illness at scale.

## Introduction

**Figure 1:**
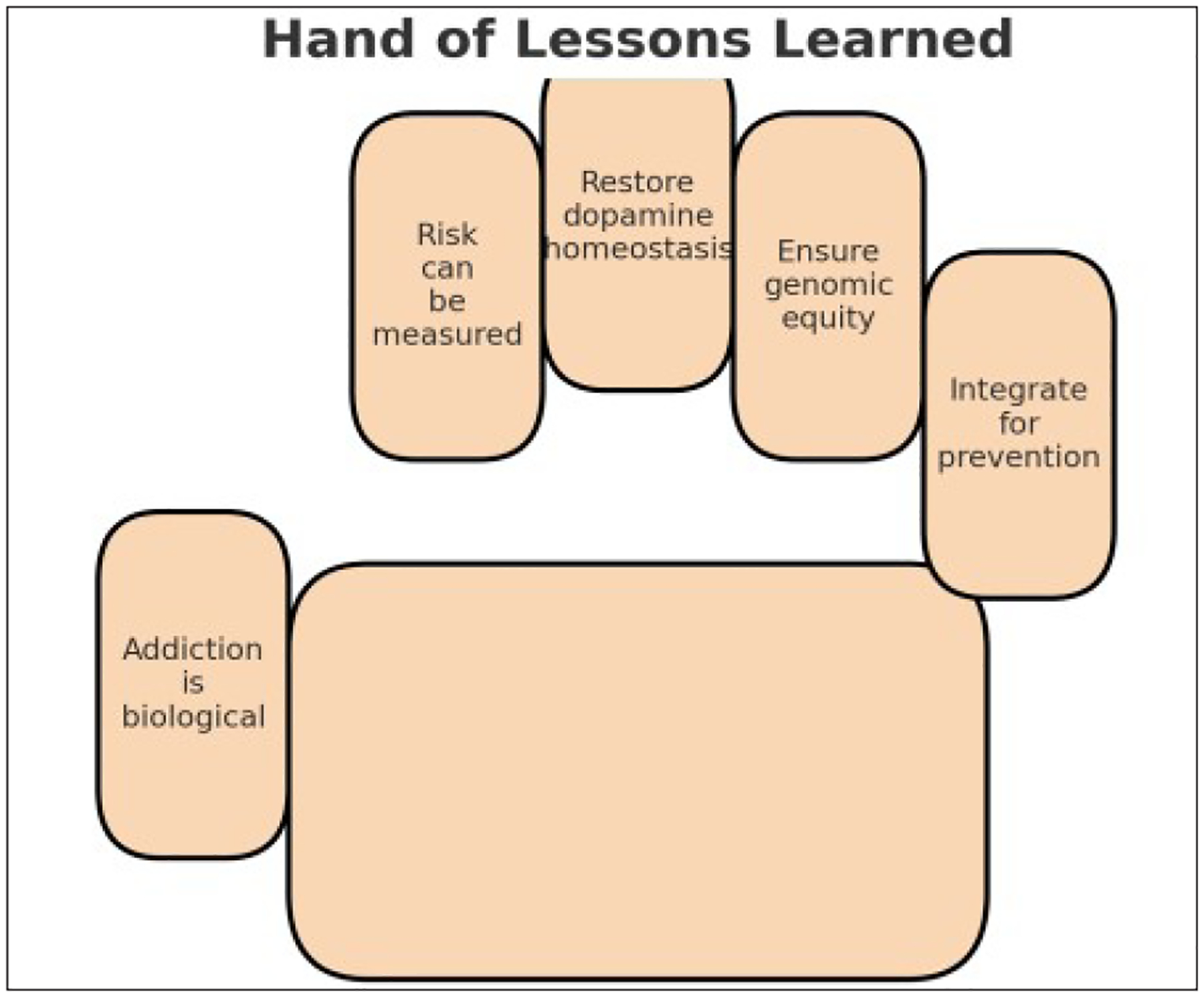
A graphical abstract illustrates what we have termed: hand of lessons learned for precision medicine in the new era of psychiatry.

Addiction neuroscience is a multidisciplinary field focused on understanding and treating both substance-related and behavioral addictions, including conditions such as eating disorders. Researchers in this area aim to uncover the neurobiological mechanisms underlying addictive behaviors and their expression across individuals and populations [1]. Over the past three decades, there has been a significant expansion in research on substance use disorders (SUD), alongside advances in our understanding of the genetic, epigenetic, and neural substrates that drive these conditions (Figure 1).

Novel methodologies, both clinical and preclinical, have been developed to investigate molecular and neurochemical changes within key neural circuits. In the context of SUD, it is crucial to acknowledge that vulnerability often stems from a combination of genetic predispositions and environmental insults, many of which are mediated by epigenetic mechanisms [2, 3]. While clinical practices still include administering opioids to treat opioid dependence, arguably treating the symptom as though it reflects an underlying opioid deficiency [4], recent findings point toward more complex neurogenetic and epigenetic pathways that influence susceptibility and resilience to addiction-related behaviors [5, 6]. For example, an impaired capacity to defer immediate gratification in favor of longer-term rewards may reflect underlying disruptions in both neural circuits and behavioral regulation [7]. A comprehensive understanding of the interaction between genetic predispositions and environmental exposures (i.e., epigenetic modulation) is essential for developing effective prevention and treatment strategies for both substance and behavioral addictions [8, 9].

Dopaminergic signaling, involving multiple neurotransmitters and second messengers, plays a central role in maintaining psychological well-being. These neurochemical interactions influence dopamine release in key brain regions, particularly the nucleus acumens, often referred to as the brain’s reward center [10]. In 1995, Kenneth Blum introduced the concept of RDS, a clinical framework describing hypo-functionality within the dopaminergic system. RDS is characterized by a diminished capacity to experience pleasure, heightened behavioral compulsivity, and maladaptive reward-seeking [11–13]. Both inherited and acquired states of hypodopaminergic have since been implicated in the development of RDS [14]. Individuals affected by this condition may engage in substance use as a compensatory strategy to temporarily restore reward signaling and alleviate dysphoria [15].

However, repeated substance use not only fails to resolve the underlying deficit but can worsen dopaminergic dysfunction over time, reinforcing the cycle of addiction and elevating physiological and psychological stress levels [16]. In addition, negative emotional states may further exacerbate RDS through epigenetic modifications, such as histone methylation, which can lead to sustained alterations in gene expression and neurobiological function [17].

### Precision genomics targeting mental illness

Long-term use of alcohol and addictive substances impairs brain networks involved in executive function, increasing vulnerability to mental illness.

In this realm, the RDS Consortium consisting of fifty scientists across the globe has made significant breakthroughs over at least 6 decades of research under the leadership of Professor Kenneth Blum [18–22].

Historically, this remarkable body of work beginning in the1970s led to the development of the first discovery of the amino acid -DL-phenylalanine, a substance that inhibits the brain opioid peptide catabolic (breakdown) system shown to reduce craving behaviors for not only alcohol but cocaine, heroin and even sugar. During these early years Blum’s group were the first to suggest that the narcotic antagonist naloxone could also block alcohol cravings as well. This formidable research led to now naltrexone’s path to Food and Drug Administration (FDA) approval to treat both alcoholism and opioid dependence. Along these lines, Blum’s team developed in the early 80’s the first commercialization of a pro-dopamine regulator called KB220 (trade names SAAVE, TROPAMINE, and PHENCAL etc.). It is noteworthy that from 1984 – 1990 commercialization of these products had a remarkable clientele consisting of over 1000 treatment centers in the USA, only to cease because of the issue regarding 35 cases of contaminated batch of tryptophan inducing eosinophilia causing scare amongst practitioners [23–27].

Flash forward to the late 80’s whereby Dr. Blum and Dr. Ernes P. Noble (former director of NIAAA) discovered the first confirmed gene for severe alcoholism -DRD2A1 allele published in JAMA in 1990. This finding quite controversial is now the most studied gene variant in all mental illness. In fact, over the many following years this variant whereby people carrying two copies unfortunately will experience a 40% loss of the required D2 receptors. To flash forward in 2025, the DRD2 and associated variants are considered the top genetic variants associated with depression, suicidal ideation and all SUD and behavioral addictions (e.g. overeating, obesity, smartphones, internet addiction etc.) [8, 26].

The term “RDS” coined by Dr. Blum in 1995 turned the mental health professionals’ heads and now boasts over 270 PUBMED listed articles supporting including 1616 for just Reward Deficiency alone. The concept of RDS has appeared in a number of medical dictionaries (e.g. Gates etc.) and is included as a featured psychological disorder in the SAGE Encyclopedia of Abnormal and Clinical Psychology (2017) [12, 13].

In the late 90’s KB220 was packaged for commercialization and the KB220 variant Phen Cal was the number one weight loss product from 1997 – 1998. To date the actual product which is patented in the USA (10,894,024 issued in 2021) and pending in Europe boasts 36 published studies (3/36 are ingredient investigation pre-clinical). The body of scientific articles is considered to be the most scientific published on any finished product in the nutrition industrial space [27].

In 2014, Blum and associates developed the GARS^®^ test. This test consists of ten genes and eleven SNPS (variants). In 2022, statistical validation of GARS in 74,566 case-control subjects was published. Currently there are 101 PubMed listed studies. Along these lines, Geneus Health, LLC., offered the first precision genomic test coupling GARS DNA results and customized KB220 variants. Most importantly, the RDS Consortium (via NIH funded studies) have published GWAS and in deep silico studies revealing the high predictability of “pre-addiction,” a major cornerstone to all mental illness. Most importantly, through the work of Alireza Sharafshah, we now have discovered the 1) Fountain of Youth gene map 2) Only five GARS predicted genes required. In fact, DRD2, DRD4, OPRMI, COMT and 5-HTTLPR has been shown to predict all mental illness, pre-addiction & dopamine dysregulation in 70 million subjects at a range of p values of 10^−16^ or 10^−17^. This indeed is a real game changer for early diagnosis of risk for all mental disorders. In the face of the opioid crisis, this five panel GARS test offers every psychiatrist, psychologist, pain specialist, neurologist, etc., the option now to prescribe an affordable early genetic predictive test [28–31].

### Pipeline

It is noteworthy that the RDS Consortiums paused to futuristic research that will involve a novel gene editing platform to “cure” RDS and even novel Biomarkers as well. Opioid overdose continues to claim over 100,000 lives annually [20]. Additionally, an estimated 800 million individuals globally exhibit addiction-related behaviors and characteristics consistent with RDS, highlighting the urgent need for innovative and preventive strategies in the management of addiction [21]. We firmly advocate for early identification of pre-addiction traits using tools such as the GARS test, a five-gene panel that offers a promising approach to primary prevention [22].

Current FDA-approved treatments for OUD include the prescription of potent opioids, which, while effective in harm reduction, carry an inherent risk of promoting dependency. Although this pharmacologic approach has merit in reducing immediate mortality and morbidity, it also underscores the necessity for non-addictive, sustainable solutions. As scientists and clinicians, we bear the responsibility of pursuing novel interventions aimed at correcting dopamine dysregulation and restoring dopaminergic homeostasis in the brain. This can be achieved through safer, non-pharmacological modalities such as neuromodulation, nutraceuticals, awareness integration therapy, cognitive therapies, and mindfulness-based interventions [32, 33].

Health equity, defined as the state in which every individual has a fair and just opportunity to attain optimal health, remains an unmet goal within the field of human genomics. To date, genomics research has failed to adequately represent the diversity of the global population, leading to disparities in both scientific understanding and clinical application. This underrepresentation poses significant limitations, perpetuating inequities and undermining the translational potential of genomic advances. Recognizing these challenges, the National Human Genome Research Institute has initiated efforts to promote equity in genomics. This includes convening domain experts to assess the current landscape and provide recommendations to bridge gaps at the intersection of genomics and health equity [34].

In the aftermath of the Human Genome Project, there were high expectations for the transformative potential of genomics to revolutionize the diagnosis, treatment, and prevention of disease. Today, we must critically assess the progress of genomic medicine. Where has the field fulfilled its promise? Where has it fallen short—and why? What unforeseen developments have emerged? Despite a slower-than-anticipated timeline, we maintain that the foundational optimism regarding genomics impact on medicine remains well placed. However, a renewed focus on the fundamental genotype-to-phenotype relationship is necessary to unlock the full potential of genomics in advancing human health and well-being [35].

The incorporation of GWAS into clinical medicine marks a pivotal evolution in healthcare. GWAS enables comprehensive examination of the human genome, producing vast amounts of sequencing data that fuel the discovery of genotype–phenotype relationships [36]. Modern bioinformatics employs advanced algorithms for variant detection and increasingly sophisticated classification models. Data science and machine learning tools, such as Python-based frameworks [37], are instrumental in processing and interpreting these data, enabling new discoveries and reinforcing existing knowledge.

In clinical practice, GWAS facilitates the development of precision medicine, allowing for tailored interventions based on a patient’s unique genetic and biochemical profile. Key areas of application include rare disease diagnostics, oncogenomic, pharmacogenomics, neonatal screening, and infectious disease genomics. A major frontier is the integration of GWAS into multi-omics approaches, promoting a systems-level understanding of human biology. Technological advancements have also led to the emergence of third- and fourth-generation sequencing methods, including long-read sequencing, single-cell genomics, and nanopore-based technologies. These cutting-edge innovations, alongside their growing application in clinical and translational research, signify a promising future for the field of genomic medicine.

## Conclusion

The evolution of addiction neuroscience has revealed that substance and behavioral addictions are not merely matters of willpower or environment, but deeply rooted in genetic, neurochemical, and epigenetic dynamics. The development of concepts like RDS, the identification of key gene variants such as DRD2, and the implementation of tools like the GARS^®^ test signify a paradigm shift toward early identification and individualized treatment of addiction risk. Importantly, this precision approach offers promising non-pharmacological strategies, such as pro-dopaminergic regulation and integrative therapies, to restore dopamine homeostasis without compounding dependency. As we confront global addiction crises, including the devastating impact of opioid overuse, the field must continue prioritizing safe, personalized, and equitable interventions. Future directions lie in expanding the reach of genomic technologies such as GWAS and multi-omics integration while addressing the systemic disparities in genomic research.
